# Rapid Formation and Rupture of Multiple Abdominal Pseudoaneurysms: A Life Threatening Case of Segmental Arterial Mediolysis

**DOI:** 10.3400/avd.cr.20-00172

**Published:** 2021-09-25

**Authors:** Ernest M Cheng, Kerry L Chen, Varsha Sharma, Juliana Yee, Mark Power, Lubomyr D Lemech, Francis Chu

**Affiliations:** 1 Department of Surgery, St George Hospital, Sydney, Australia; 2 St George Clinical School, University of New South Wales, Sydney, Australia; 3 Department of Radiology, St George Hospital, Sydney, Australia; 4 Department of Vascular Surgery, St George Hospital, Sydney, Australia

**Keywords:** segmental arterial mediolysis, coil embolization, pseudoaneurysms

## Abstract

We present a 62-year-old gentleman with rapidly forming abdominal pseudoaneurysms due to segmental arterial mediolysis (SAM). With rupture of his pseudoaneurysms, he underwent angiography and successful coil embolisation. In this case, we demonstrate the potential for rapid progression of pseudoaneurysms in SAM, with the need for prompt diagnosis and urgent endovascular intervention.

## Introduction

Segmental arterial mediolysis (SAM) is an uncommon but critical condition with up to a 60% mortality rate in the acute phase from catastrophic hemorrhage. It is a non-inflammatory, non-atherosclerotic vasculopathy with segmental lysis of the tunica medial layer of abdominal arteries.^1)^ SAM most commonly affects coeliac, mesenteric and renal arteries, and its presentation varies from chronic mesenteric ischaemia to acute life-threatening haemorrhage thus posing both a diagnostic and therapeutic challenge.^1,2)^ We report a case of SAM in a 62-year-old gentleman presenting with rapid formation and rupture of pseudoaneurysms who was successfully treated with early coil embolisation.

## Case Report

**Figure 1:**
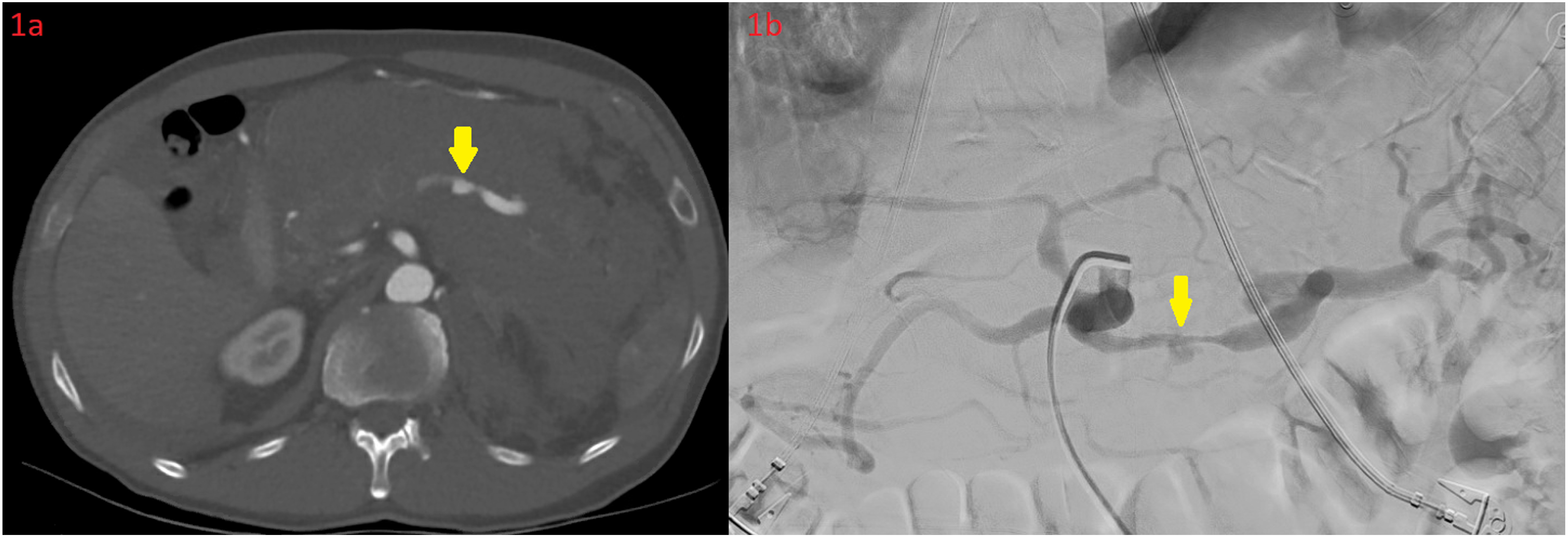
Fig. 1 (**a**) Computed tomography angiogram demonstrating splenic artery pseudoaneurysm surrounded by retroperitoneal haemorrhage. (**b**) Angiography demonstrating proximal splenic artery pseudoaneurysm.

**Figure 2:**
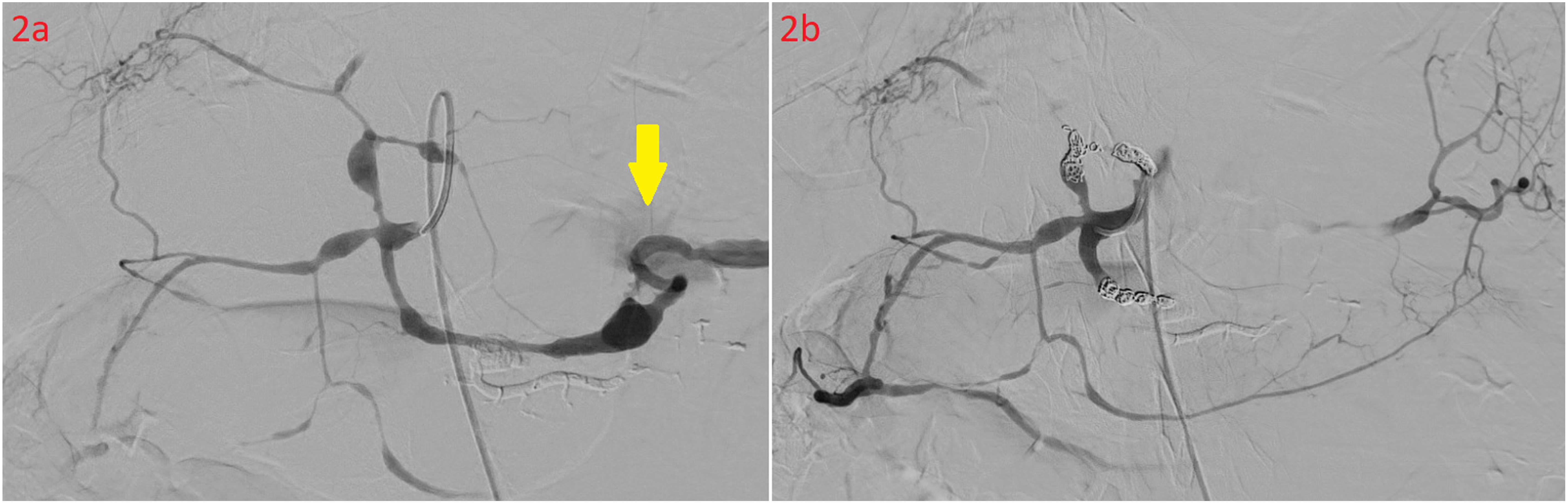
Fig. 2 (**a**) Before embolization: active bleeding from the distal splenic pseudoaneurysm, new left gastric artery pseudoaneurysm and previous embolization site of proximal splenic artery. (**b**) Post-embolisation of left gastric artery and distal splenic pseudoaneurysms.

**Figure 3:**
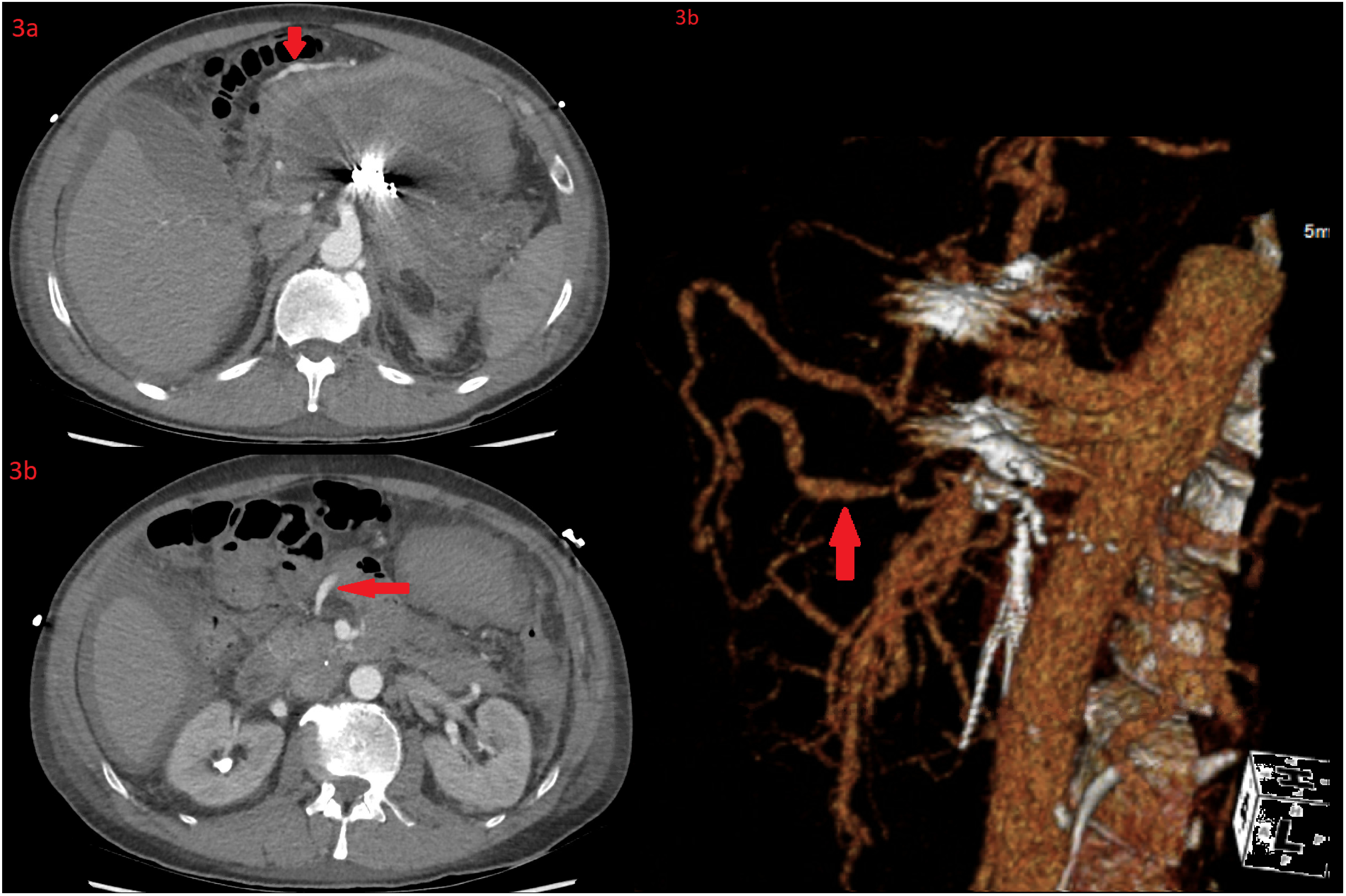
Fig. 3 (**a**) “String of beads” radiographic findings seen at the gastroepiploic artery. (**b**) Mid superior mesenteric artery dilatation on computed tomography. (**c**) 3-Dimensional reconstruction of the mid superior mesenteric artery dilatation.

A 62-year old gentleman presented with sudden onset, severe generalised abdominal pain. He had normal vitals signs but on examination demonstrated generalised peritonism. Biochemistry showed lactataemia of 4.2 mmol/L but was otherwise unremarkable. Computer tomography (CT) imaging of his abdomen was equivocal for an early superior mesenteric artery dissection. Spontaneous and complete resolution of the patient’s symptoms occurred within an hour of presentation and he was admitted for monitoring. Twenty-four hours later the patient experienced the same severe, sudden onset, abdominal pain accompanied by hypotension and syncope. A CT mesenteric angiogram demonstrated two pseudoaneurysms arising from the splenic artery with associated rupture of the proximal pseudoaneurysm and retroperitoneal haemorrhage (**Fig. 1**). The proximal pseudoaneurysm was embolised by interventional radiology. Four hours later, the patient became haemodynamically unstable with a significant haemoglobin drop (24 g/L) requiring massive transfusion. Repeat CT mesenteric angiogram demonstrated new left gastric, short gastric and distal splenic pseudoaneurysms with rupture of the distal splenic pseudoaneurysm. Coil embolisation was performed on the haemorrhaging distal splenic artery pseudoaneurysm and preventatively on the left gastric artery pseudoaneurysm (**Fig. 2**). The patient had no further bleeds and was monitored in intensive care unit. Subsequent CT angiogram demonstrated further aneurysmal dilatations along the gastroepiploic artery, middle colic branch of the superior mesenteric artery (SMA) and left common iliac artery (**Fig. 3**). A dissection flap at the mid SMA was also noted, however, this was present on previous CT imaging and unchanged. All vasculitis, thrombophilic and infective screens were negative. This patient’s clinical presentation combined with the radiological findings were consistent with SAM.

His recovery was complicated by multiple segmental and subsegmental pulmonary embolisms requiring insertion of an inferior vena cava filter and therapeutic anticoagulation. However, he had no further haemorrhagic events. He presented 3 months later after falling off a horse and suffered 6 rib fractures on his left side. Despite the significant force from the fall, abdominal imaging demonstrated stable aneurysmal dilatations and the patient demonstrated no further abdominal bleeding episodes. He was followed up 6 months after the event with no further bleeding.

## Discussion

SAM is a rare vascular disorder with unknown aetiology first described by Slavin and Gonzalez-Vitale in 1976.^1)^ It characteristically affects medium to large gastrointestinal arteries and causes degeneration of the tunica media without inflammatory, atherosclerotic or hereditary components.^3)^ Although there are no proven risk factors, hypertension is commonly associated with SAM in the literature.^2)^ There are currently no known precipitating factors for an acute episode of the disease and patients typically present with sudden abdominal pain secondary to dissection, ischaemia or haemorrhage.^2,4)^ SAM poses a diagnostic challenge as clinical presentations can vary from a brief episode of abdominal pain with stable disease to significant haemodynamic instability from haemorrhage. As such, the natural history of SAM is poorly understood and the estimated mortality rate of SAM varies from 0 to 60%.^2,5,6)^ Diagnosis of SAM may not require histopathological confirmation, as CT findings of arterial dissection, fusiform aneurysms, wall thickening and “string of beads” appearance affecting abdominal visceral arteries are often sufficient for diagnosis.^2)^ Kalva et al. described a non-invasive diagnostic criteria for SAM. Its criterion is based on clinical and radiological findings described above and the absence of congenital predisposition for dissection (e.g. Ehlers-Danlos, Marfan, Leoys-Dietz) and a low suspicion of alternative arteriopathies (e.g. fibromuscular dysplasia, collagen vascular disorders or arteritis). Negative inflammatory markers such as antinuclear antibodies and antineutrophil cytoplasmic antibodies contributed to the diagnosis of SAM.^7)^ However, tissue diagnosis, if obtainable, can assist in differentiating mimics of SAM include anti-neutrophil cytoplasmic autoantibody-associated vasculitis, polyarteritis nodosa, C and mycotic aneurysmal infections.^8)^ In cases where the early phase of SAM when the diagnosis is unclear, a combination of tissue diagnosis, vasculitis biomarkers and infection screens can be useful in distinguishing similar arteriopathies.^2)^

Treatment for SAM in the acute setting includes emergent endovascular intervention, open surgery or medical management.^2,5,8)^ Shenouda et al. reviewed 85 cases of SAM with 24 cases utilising endovascular intervention and 36 cases undergoing emergency open laparotomy. Open surgery was associated with poorer outcomes with 9% mortality compared to 0% in those undergoing endovascular treatment.^8)^ However, open surgery is valuable in the event of failed endovascular intervention or in the elective setting for vessel ligation for known stable pseudoaneurysms.^8,9)^ Alternatively, patients who survived the acute phase of the disease can be managed medically with anti-hypertensives, antiplatelets or anticoagulation in combination with serial CT imaging.^5)^ Fortunately, disease regression and stability were observed in many patients after resolution of their initial acute episode. Several studies have concluded after the acute phase, majority of patients demonstrated regression or stability of disease.^5,10,11)^

## Conclusion

Vascular surgeons should be aware of SAM as a differential for patients presenting with acute abdominal pain. SAM as a life-threatening condition requiring the need for early angiography and embolization via interventional radiology, rather than diagnostic open exploratory laparotomy. This case contributes to our understanding of the variability in SAM’s clinical presentation and knowledge of its potential to rapidly propagate pseudoaneurysms at risk of rupture. It emphasises the need for vigilance when approaching abdominal pain with initially unclear aetiology.
